# Response: Commentary: Age-related neurodegenerative disease research needs aging models

**DOI:** 10.3389/fnagi.2016.00044

**Published:** 2016-03-04

**Authors:** Ian P. Johnson

**Affiliations:** Discipline of Anatomy and Pathology, The University of AdelaideAdelaide, Australia

**Keywords:** aging, neurodegenerative diseases, models, biological, frailty index, commentary

In response to my recent article (Johnson, 2015), Wallace and Howlett have provided a thoughtful commentary pointing out that aging might be defined not just by the passage of time, but by the accumulation of defects in multiple regions throughout the organism that can be represented by a frailty index (Wallace and Howlett, 2016). It occurs to me that this approach might help overcome two major barriers to aging research: (i) the time taken for animals to age, and (ii) the fact that animals tend to die as they age. In our research on aging (24 m-old) ad-libitum fed rats, we found approximately 50% of animals died before the age of 24 m. In contrast, caloric-restricted rats did not die before they reached 24 m, did not show typical age-related pathologies, and had motoneurones that responded differently to injury compared to age-matched ad-libitum-fed rats (Aperghis et al., 2003; Johnson and Duberley, 1998). For 10–15-years-old *ad-libitum* fed cats we also found increasing health problems with age, but no evidence that the spinal motoneurones in these aging cats were any more vulnerable to injury than those in 1–2-years-old cats (Johnson et al., 1991). It would be interesting to know if the creation in younger animals of age-related defects, such as those associated with metabolic syndrome or changes in inflammatory status, also causes nervous system pathologies typical of aging. Such models may well be quicker and cheaper than aging animals. They might also remove the criticism that results obtained from the study of aging animals are unrepresentative because they are based on the “survivors.” The use of a frailty index to determine age and perhaps create artificially aged models is an exciting concept, notwithstanding the possibility that the largely post-mitotic population of neurones in the nervous system may respond differently to cells in the rest of the organism.

## Author contributions

The author confirms being the sole contributor of this work and approved it for publication.

### Conflict of interest statement

The author declares that the research was conducted in the absence of any commercial or financial relationships that could be construed as a potential conflict of interest.
